# Folic Acid Supplementation during Pregnancy and Its Association with Telomere Length in Children at Four Years: Results from the INMA Birth Cohort Study

**DOI:** 10.3390/nu15194303

**Published:** 2023-10-09

**Authors:** Fanny Petermann-Rocha, Desirée Valera-Gran, Daniel Prieto-Botella, Dries S Martens, Sandra Gonzalez-Palacios, Isolina Riaño-Galán, Mario Murcia, Amaia Irizar, Jordi Julvez, Loreto Santa-Marina, Adonina Tardón, Jordi Sunyer, Jesús Vioque, Tim Nawrot, Eva-María Navarrete-Muñoz

**Affiliations:** 1 Centro de Investigación Biomédica, Facultad de Medicina, Universidad Diego Portales, Santiago 8370109, Chile; fanny.petermann@udp.cl; 2 Department of Surgery and Pathology, Miguel Hernandez University, 03550 Alicante, Spain; dprieto@umh.es (D.P.-B.); enavarrete@umh.es (E.-M.N.-M.); 3 Grupo de Investigación en Terapia Ocupacional (InTeO), Miguel Hernandez University, 03550 Alicante, Spain; 4 Instituto de Investigación Sanitaria y Biomédica de Alicante, Universidad Miguel Hernández (ISABIAL-UMH), 03010 Alicante, Spain; sandra.gonzalezp@umh.es (S.G.-P.); vioque@umh.es (J.V.); 5 Centre for Environmental Sciences, Hasselt University, 3590 Hasselt, Belgium; dries.martens@uhasselt.be (D.S.M.);; 6 CIBER de Epidemiología y Salud Pública (CIBERESP), Instituto de Salud Carlos III (ISCIII), 28029 Madrid, Spain; rianoisolina@uniovi.es (I.R.-G.); mariomurhi@gmail.com (M.M.); amaia.irizar@ehu.eus (A.I.); jordi.julvez@urv.cat (J.J.); l-santa@euskadi.eus (L.S.-M.); atardon@uniovi.es (A.T.); jordi.sunyer@isglobal.org (J.S.); 7 Unidad de Epidemiología de la Nutrición (EPINUT), Departamento de Salud Pública, Historia de la Ciencia y Ginecología, Universidad Miguel Hernández (UMH), 03550 Alicante, Spain; 8 Instituto de Investigacion Sanitaria del Principado de Asturias (ISPA), University of Oviedo, 33003 Oviedo, Spain; 9 Pediatrics Unit, Central University Hospital of Asturias, 33011 Oviedo, Spain; 10 Epidemiology and Environmental Health Joint Research Unit, FISABIO-Universitat Jaume I-Universitat de València, 46020 Valencia, Spain; 11 Servicio de Análisis de Sistemas de Información Sanitaria, Conselleria de Sanitat, Generalitat Valenciana, 46010 Valencia, Spain; 12 Department of Preventive Medicine and Public Health, Faculty of Medicine and Nursing, University of the Basque Country, 48940 Leioa, Spain; 13 Group of Environmental Epidemiology and Child Development, Biodonostia Health Institute, 20014 San Sebastian, Spain; 14 Clinical and Epidemiological Neuroscience Group (NeuroÈpia), Institut d’Investigació Sanitària Pere Virgili (IISPV), 43204 Reus, Spain; 15 Instituto de Salud Global (ISGlobal) de Barcelona Campus MAR, Parc de Recerca Biomèdica de Barcelona (PRBB), 08003 Barcelona, Spain; 16 Health Department of Basque Government, Sub-Directorate of Public Health of Gipuzkoa, 20013 San Sebastian, Spain; 17 Instituto Universitario de Oncología Del Principado de Asturias (IUOPA), Departamento de Medicina, University of Oviedo, 33006 Oviedo, Spain; 18 Departament de Ciències Experimentals i de la Salut, Universitat Pompeu Fabra, 08002 Barcelona, Spain

**Keywords:** folic acid supplementation, telomere length, pregnancy, child

## Abstract

This study examined the association between folic acid supplements (FAs) during different periods of pregnancy and offspring telomere length (TL) at age four in 666 children from the INMA study. FAs were self-reported using food-structured questionnaires during three periods of pregnancy (the first three months of pregnancy, from month fourth onward, and the whole pregnancy). For each period, the average daily dosage of FAs was categorised into (i) <400 μg/d, (ii) ≥400 to 999 μg/d, (iii) ≥1000 to 4999 μg/d, and (iv) ≥5000 μg/d. Leucocyte TL at age four was measured using quantitative PCR methods. Multiple robust linear log-level regression models were used to report the % difference among FA categories. During the first period, and compared with children whose mothers were classified in the reference group (<400 μg/d), children whose mothers took higher dosages of FAs showed shorter TL at age four (≥5000 μg/d). When the first and the second periods were mutually adjusted, children whose mothers self-reported ≥5000 μg/d during the first period of pregnancy had a statistically significant shorter TL than their counterparts (% difference: −7.28% [95% CI: −14.42 to −0.13]). Similar trends were observed for the whole period of pregnancy. When the analysis was stratified by sex, the association was more evident in boys (% difference: −13.5% [95% CI: −23.0 to −4.04]), whereas no association was observed in girls. This study suggests that high dosages of FAs in the first pregnancy period may be associated with a shorter TL in children at age four, particularly among boys. Further studies should confirm these results.

## 1. Introduction

Folate, an essential micronutrient, is naturally abundant in a wide variety of foods, such as broccoli, cabbage, cauliflower, fruits, and nuts. Its synthetic counterpart, folic acid (FA), is incorporated into fortified food items and vitamin supplements because of its remarkable stability compared to the naturally occurring form. Folate plays a pivotal role in DNA replication during cell synthesis and division [1]. This micronutrient has been widely investigated for its role in DNA methylation and its ability to mitigate the risk of neural tube defects (NTDs) during pregnancy. Since 1998, the World Health Organisation has recommended an intake of FAs of 400 µg per day (µg/d) from conception until week 12 of pregnancy, not exceeding 1000 μg/d, to reduce the NTD risk [2]. However, there is concern regarding the potential effect of high maternal supplement dosages on child health outcomes associated with unmetabolised FA identified [3]. Previous studies highlighted that maternal supplementation with ≥1000 μg/d may negatively impact birth outcomes and children’s cognitive development later in life [4,5,6,7]. Moreover, recent studies have also suggested that high maternal FA supplements (FAs) dosages might also influence telomere length (TL) in children [8,9].

Telomeres are nucleoprotein structures at the end of linear chromosomes (TTAGGG) [10]. They serve a critical role by safeguarding against illicit DNA repair, degradation, and end-to-end fusion, ensuring the preservation of physical integrity in linear eukaryotic chromosomes, while also facilitating meiotic segregation and nuclear organisation [10]. Telomeres naturally shorten over time, yet under exogenous stressors, they are prone to quicker shortening [11,12]. Accumulating evidence has shown that shorter telomeres have been linked to many age-related disorders and a higher mortality rate [12]. Despite being recognised as an ageing biomarker, recent insights have shown that childhood is a stage of life marked by dynamic biological processes and a significant dependence on genetic and environmental factors, making it a pivotal period for understanding telomere attrition [13]. Previous studies have proposed that the well-established environmental conditions associated with TL during adulthood may have less impact on TL than conditions related to childhood [13,14]. For instance, Martens et al. identified that TL at four years has a stronger correlation with TL from the placenta (r = 0.60) than with TL in adulthood (r = 0.46) [15]. However, the study of telomeres during childhood remains limited and further research on childhood telomere determinants is required [13].

Folate, or FA, plays a direct role in DNA methylation, a critical process in gene transcription. This is a pivotal source of one-carbon units that affect reaction efficiency for crucial cellular pathways, such as DNA, RNA, and protein methylation, as well as DNA synthesis and maintenance [1]. In this context, an imbalance in this micronutrient may lead to the breakage, shortening, and alteration of telomere function [3]. Presumably, folate deficiency may cause elevated uracil integration and reduced DNA methylation within telomeres, potentially influencing their structural integrity and interactions with associated proteins and raising queries about folate’s involvement in nucleoplasmic bridges and chromosome fusions affecting telomeric regions [16,17]. Considering these concerns, alongside growing awareness of the potential detrimental effects of unmetabolised FA, we hypothesised that there could be a connection between excessive maternal FA intake and a reduction in TL. The unmetabolised FA might disrupt the balance of DNA methylation within telomeres, leading to structural changes and functional alterations in these crucial genomic regions. However, the currently available evidence regarding the effect of FAs during pregnancy on TL remains insufficient for a comprehensive assessment of this potential association. For instance, a randomised clinical trial (RCT) in Ghana did not show evidence between nutritional supplementation during gestation and TL at 4–6 years [8], while a cohort study in women from the US concluded that maternal total folate concentration in early pregnancy was associated with newborn cord blood TL [9]. Thus, considering the role of FA during pregnancy and the reduced information on the effect over TL, a systematic review regarding maternal diet and offspring TL recommended further exploring the association between these two variables [18]. Therefore, this study aimed to investigate the association between FAs during different periods of pregnancy and offspring TL at age four.

## 2. Materials and Methods

### 2.1. Study Design and Study Population

This research was conducted using data from the INMA (INfancia y Medio Ambiente; Environment and Childhood) birth cohort study. The primary objective of the INMA initiative is to explore the influence of environmental factors throughout pregnancy and infancy and their impact on the growth and development of children. More details about the INMA project are available online at https://www.proyectoinma.org/ (accessed on 24 March 2023).

**Figure 1:**
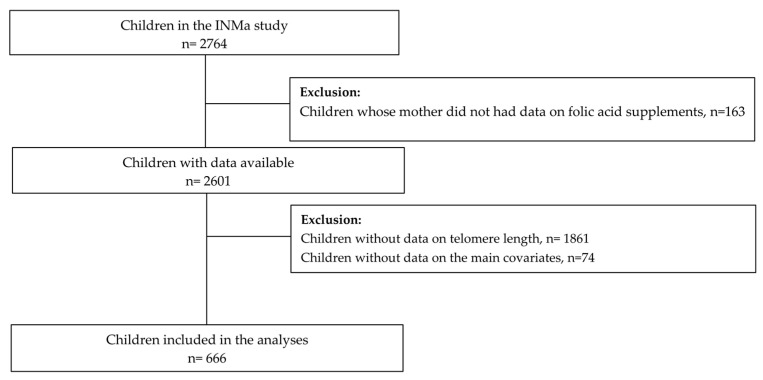
Flowchart of the study population describing the selection process.

In summary, this study recruited pregnant women from the general population within the time frame of 2004 to 2008 in four distinct regions of Spain, namely Asturias, Gipuzkoa, Sabadell, and Valencia. Inclusion criteria comprised the following: women aged ≥16 years, expecting a singleton pregnancy, not undergoing assisted conception, planning to give birth at the designated hospital, and having no communication difficulties. Out of the initial sample of 2764 participants, a total of 666 children had complete data available regarding the exposure variable (FAs), the primary outcome (TL at four years), as well as relevant covariates, and thus, they were included in the subsequent analyses (Figure 1).

### 2.2. Folic Acid Supplementation

FAs and various vitamins and minerals containing FA were obtained by administering a dietary questionnaire designed to capture food-related information. In addition, supplementary information regarding brand names, composition, daily dosage, and the timing of consumption was gathered, as previously detailed in another study [4,5]. This questionnaire was first interviewer-administered to pregnant women at 10–13 weeks to estimate the consumption of FAs from preconception (approximately three months before conception) to the third month of pregnancy; and then at 28–32 weeks to estimate FA supplementation from the fourth to the seventh months of pregnancy. To estimate the consumption of FAs, we first quantified each woman’s monthly FA supplementation. Then, the monthly consumption of FA was summed and averaged by the number of months for each period of pregnancy (first period, from three months before conception to the third month of pregnancy; second period, from the fourth to the seventh month of pregnancy) and for the whole pregnancy (i.e., from preconception to the seventh month).

For each period and the whole pregnancy, the daily average of FA intake was categorised into the following categories: <400 μg/d; ≥400 to 999 μg/d; ≥1000 to 4999 μg/d; and ≥5000 μg/d.

### 2.3. Leucocyte Telomere Length Measurement

Leucocyte TL at age four served as the metric for TL measurement. These measurements were obtained from individuals in Gipuzkoa, Sabadell, and Asturias, with an average age of 4.4 years and a standard deviation of 0.2 years. Blood samples were collected during clinical examinations and were properly stored in EDTA tubes. DNA extraction from the blood samples was conducted using different kits depending on the location: the Flexigen AGKT-WB-640 kit (Qiagen, Singapore) for Gipuzkoa samples; the Chemagen kit (Perkin Elmer, Waltham, MA, USA) for Sabadell samples; and the QIAamp DNA Mini Kit (Qiagen) for Asturias samples.

Technical details of leucocyte TL measurements using qPCR are available elsewhere [15]. Telomere measurements were performed in triplicate, and during each run, a 6-point serial dilution of a pooled DNA sample (consisting of 12 DNA samples) was included to assess the efficiency of quantitative polymerase chain reaction (qPCR) for both telomere (T) and single-copy gene (S) assays. The efficiency for the T runs was determined to be 107%, with R2 values ranging from 0.994 to 0.999, while the S runs exhibited an efficiency of 97%, with R2 values ranging from 0.995 to 0.999. To calculate relative leukocyte TL for each cohort, qBase software (Biogazelle, Zwijnaarde, Belgium) was employed. Within the qBase software (3.4 version; CellCarta 2008-2023), TL was computed as a calibrated normalised relative quantity (CNRQ) [19]. The latter was achieved by first calculating the RQ based on the delta-Cq method for T and S obtained Cq values, using target-specific amplification efficiencies. Given that the selection of a calibrator sample can significantly impact the error associated with the ultimate relative quantities due to potential measurement variations in the calibrator, we conducted normalisation by aligning the quantification values of all analysed samples within each cohort to the arithmetic mean. This normalisation procedure yielded the normalised relative quantity (NRQ). Additionally, since samples from each cohort underwent measurement across multiple qPCR plates, we employed eight inter-run calibrators (IRCs) to calculate an additional correction factor. This factor was applied to mitigate run-to-run differences, ultimately yielding the telomere to single-copy gene ratio (CNRQ). The mathematical formulas required for calculating RQ, NRQ, and CNRQ were sourced from Hellemans et al.’s work in 2007 [20]. On each run, the reliability/accuracy of the applied protocol was assessed by calculating the intraclass correlation coefficients (ICC) of triplicate measures for T values (ICC: 0.957 [95% CI: 0.954 to 0.96], *p* < 0.0001), S values (ICC: 0.968 [95% CI: 0.965 to 0.97], *p* < 0.0001), and T/S ratios (ICC: 0.925 [95% CI: 0.918 to 0.93], *p* < 0.0001), using the ICC R-code provided by the Telomere Research Network [21]. In addition, based on the 8 IRCs’ run over, all the qPCR plates on an inter-assay ICC were calculated (ICC: 0.898 [95% CI: 0.77 to 0.948], *p* < 0.0001). Based on the standard curves, qPCR efficiency for T runs was, on average, 107.

### 2.4. Covariates

Mother’s age during pregnancy (in years), parity (number of previous children, classified as 0 or ≥1), smoking mother before pregnancy (yes or no), and mother’s education (classified as low [primary], middle [secondary] and high [university]) were self-reported. Alcohol (g/d), folate (μg/d), and total energy intake (in kilocalories/day) were collected using an adapted and validated food-frequency questionnaire for pregnant women in the first and second period of pregnancy [22]. Regarding offspring data, sex (female or male), blood extraction date (the day when telomere information was collected and then codified as the season of extraction), sleep (reported by the caregivers in hours per day), ultra-processed food intake according to the NOVA classification in grams/day [23], adherence to a relative Mediterranean diet score (rMED, based on the Buckland et al. index as it is described elsewhere [24]), and television (TV) time (reported by the caregivers regarding the total hours during the week and weekend) were the covariates included from the children at age four in the analyses.

### 2.5. Statistical Analyses

Descriptive characteristics by each area included (i.e., total, Asturias, Gipuzkoa, and Sabadell) are presented as median with their respective interquartile range for quantitative variables. Categorical variables were conveyed through frequency counts alongside their respective percentages. To assess the distribution of continuous variables, the Lilliefors-adjusted Kolmogorov–Smirnov test was employed, and subsequent comparisons were made using ANOVA, Kruskal–Wallis, and Chi-squared tests as appropriate.

Main models were built using covariates with *p* < 0.2 in the descriptive analysis. A backward elimination procedure was applied to select the statistically significant covariates with a *p* < 0.10. However, these covariates were held as adjustment variables in the models if they modified the magnitude of the main effect by ≥10%, regardless of their statistical significance. Associations between FA categories and TL at age four were investigated using multiple robust linear regression models, through log-level regression models, where the TL was log10-transformed. Therefore, the results are reported as a % difference and their respective 95% CI. A meta-analysis was conducted to explore cohort heterogeneity to obtain combined estimates. The heterogeneity was quantified using *I*^2^ statistics. According to the heterogeneity observed, random (*I*^2^ > 50%) or fixed effects were applied (*I*^2^ ≤ 50%). Participants in the lowest FA category (<400 μg/d) were used as the references for all periods.

All analyses were adjusted for the mother’s age, parity, smoking status, mother’s education level, intake of alcohol, folate intake, total energy intake (according to each of the periods included: first, second, or all), sex of the child, and blood extraction date (season), as well as sleep, ultra-processed food intake, rMED, and TV time of the child at age four. Moreover, further analysis was performed where the FAs in the first and the second periods were mutually adjusted. These potential confounding factors were selected based on the previous literature [25].

Finally, to investigate whether the association differed by sex, the analyses were repeated and stratified by sex (girls and boys). A *p* interaction term was calculated for each cohort. R 4.0.5 and Stata 17 were used to perform the statistical analyses. A *p*-value lower than 0.05 was considered statistically significant.

### 2.6. Ethic Declarations

The regional Ethical Committees (Medical Assistance Municipal Institute, Sabadell; Central University Hospital of Asturias, Asturias; and Donostia Hospital, Gipuzkoa) approved the INMA birth cohort study. Written informed consent was obtained from all participants. This study complies with the Helsinki Declaration for Human Studies.

## 3. Results

### 3.1. Cohort Characteristics

Six hundred sixty-six children with data available for the exposure, the outcome, and covariates were finally included (Figure 1). Sociodemographic and lifestyle characteristics of the population included in the study by area of origin are presented in Table 1. Overall, a higher proportion of children were from Asturias or Sabadell, while just 19.8% were from Gipuzkoa. Except for Gipuzkoa, a greater proportion of children were boys and had their blood extracted in Winter or Spring. Caregivers from Sabadell reported their children had more hours per week watching TV, a higher UPF intake, and a lower rMED. Concerning their mothers’ characteristics during pregnancy, women from Sabadell were statistically younger than the other cohorts, were more likely to smoke before pregnancy, and had lower educational levels. Women from Gipuzkoa had the highest proportion of university degrees and the lowest smoking prevalence before pregnancy. Regarding food and nutrients, Gipuzkoa women had the highest folate intake during the first and second periods, while the lowest was observed in Sabadell. Even if women from Gipuzkoa also self-reported the highest energy intake during the first period compared with the other two cohorts, they reduced their energy intake during the second period and showed the lowest overall intake during the whole pregnancy period. More information about the general mothers’ and children’s cohort characteristics during pregnancy and at age four can be found in Table 1.

### 3.2. Associations between FA Supplementation and TL

Associations between FA categories and TL are shown in Table 2. During the first period, and compared with children whose mothers were classified in the reference group (<400 μg/d), those whose mothers took FAs with dosages between 400 and 999 μg/d and 1000 to 4999 μg/d had 2.70% (95% CI: −5.99 to 11.4) and 0.23% (95% CI −3.19 to 3.65) longer telomeres, respectively. However, those children whose mothers had an FA intake over 5000 μg/d had shorter TL than their counterparts (% difference: −6.07 (95% CI −12.6 to 0.46). Similar trends to those observed in the first period were identified throughout the whole period. Yet, none of these associations were statistically significant (Table 2).

When the first and the second periods were mutually adjusted, children whose mothers self-reported FAs dosages ≥ 5000 μg/d during the first period of pregnancy had statistically significant shorter TL than those children whose mothers self-reported taking dosages < 400 μg/d (% difference: −7.28 [95% CI: −14.42 to −0.13]) (Table 2). The latter results were homogenous among the cohorts included (*I*^2^ = 0.0%).

### 3.3. Associations between FA Supplementation and TL by Sex

When the associations were stratified by sex, similar trends in associations were identified among girls (Table 3). Even if the associations were not statistically significant in any of the periods and FA categories, a trend in shorter TL was identified in girls whose mothers had FAs intakes ≥ 5000 μg/d during the first period compared to the reference group. Boys, instead, showed a clear trend (Table 4). For instance, boys whose mothers self-reported consuming FAs with dosages between 1000 and 4999, as well as those with ≥5000 μg/d, had −0.20% (95% CI: −4.47 to 4.07) and −11.3% (95% CI: −19.4 to −3.22), respectively, had shorter TL than those children whose mothers were in the reference group. When this association was adjusted for the second period, boys whose mothers were in the highest intake category had even shorter TL; this result was statistically significant (% difference: −13.5 [95% CI: −23.0 to −4.04]) (Table 4). Moreover, children whose mothers self-reported consuming FAs with an intake of between 1000 and 4999 showed the shortest TL both in the general model and the model adjusted for the first pregnancy period (% difference main model: −7.98 [95% CI: −15.4 to −0.53] and % difference mutually adjusted model: −7.97 [95% CI: −15.9 to −0.04]). No other significant association was identified. 

## 4. Discussion

Using data from the INMA cohort study, we identified that children whose mothers took high dosages (≥5000 μg/d) of FAs during the first period of pregnancy, as well as those in the whole period, were more likely to have shorter TL at age four than those children whose mothers were in the lowest category of FAs during these periods (<400 μg/d). The findings seen during the first period were consistent when the analysis was further adjusted for FAs during the second period. Considering that FA is essential in maintaining nuclear and mitochondrial integrity, the observed results during the first period emphasise the role of FAs during this critical stage, highlighting the impact of environmental factors in the early foetal stage over DNA and, therefore, TL.

To our knowledge, few studies have investigated the association between maternal FAs during pregnancy and TL in children. The present study is the first to identify a negative association between a high dosage of FAs and TL in the periconceptional period and during the whole pregnancy. In contrast to our findings, an RCT of 1014 pregnant women who received lipid-based nutrient supplements, iron, and FA during gestation, or multiple micronutrients during pregnancy and the first six months postpartum, found that their children at ages 4–6 did not have differences by supplementation group in their TL [8]. This discrepancy may mainly be attributed to the fact that the FA supplementation pattern of pregnant women from our study differed greatly from the latter study. While all the women of the RCT took a daily dose of 400 μg/d of FA, the mean daily dosages of FAs used by women in our study varied greatly between <400 μg and ≥5000 μg. Moreover, the RCT followed a specific supplementation protocol, while our study examined real-world supplementation patterns among pregnant women. This variation in supplementation approaches reflects the diversity of FA intake in the general population, offering insights into potential effects under various consumption scenarios. Another important aspect is that participant demographics, geographical locations, and dietary habits can vary between studies. Our study harnessed data from the INMA cohort, a sample from Spain, while the RCT was conducted in Ghana. These notable population differences are highly likely to have contributed to the divergent outcomes observed between the two studies. In contrast, a prospective study following 119 pregnant women since week 9 identified that a 10 ng/mL increase in total folate serum concentrations (including supplements) was associated with an increase in the median TL of newborns, even after adjusting for confounding factors [9]. However, although pregnant women in this study were folate-supplemented, this study did not provide information about the timing and dosage of FA supplementation. Moreover, this study included a small sample size and did not investigate what may happen with the TL of children later in life, as we did in our study. Therefore, our results are not comparable. Hence, our study not only answers our research question but also provides a unique opportunity to fill gaps in the current literature using an exhaustive list of potential confounders.

The statistical association with FAs at intakes ≥ 5000 μg/d was only observed during the first period, while a nonsignificant association was still noticeable in the second period. However, this association became more pronounced (% difference −13.5%) and nearly reached statistical significance for children whose mothers consistently consumed such high dosages throughout the entire pregnancy (*n* = 21). Nevertheless, it is important to interpret this finding cautiously due to the presence of heterogeneity in this association (*I*^2^ = 69.4). One potential explanation for the lack of observed effects in the second period could be related to the timing of FA exposure. TL dynamics may vary depending on the developmental stage of the foetus and the rate of cell division occurring during specific periods of pregnancy [25]. The first period, which encompasses the periconceptional period and early pregnancy, is a critical time for DNA synthesis and cell proliferation, in which FA plays a vital role. High FA intake during this period might impact TL because of its involvement in DNA synthesis and repair processes. In contrast, the second pregnancy period may involve different biological processes, with cell division rates and DNA synthesis potentially being less influenced by FA intake. It is also worth considering that TL regulation is a complex process influenced by multiple genetic and environmental factors [12,13,25]. The specific mechanisms that govern the relationship between FA intake and TL in different pregnancy periods might be multifaceted and require further investigation. Therefore, while we observed significant associations in the first period, the lack of observed association in the second period may be attributed to the unique timing and biological processes involved during each pregnancy period. However, we acknowledge that our findings must be interpreted with caution, and additional research is required to acquire a complete understanding of these mechanisms.

When the analyses were stratified by sex, boys—but not girls—whose mothers were in the same category of FAs (≥5000 μg/d) were those with the shortest TL at age four. This is not surprising, considering that a previous study found that girls had longer relative TL than boys at age four [26]. Even if the reason for these sex differences is still unclear, a likely explanation based on the effect of sex hormones has been recently provided [27]. Presumably, according to this study, higher telomerase activity in female embryonic cells before random inactivation of a DKC1 allele could be responsible for longer telomeres in females compared to male embryos before embryo implantation. Furthermore, the longer telomeres in females’ stem cells, endothelial cells, and lymphocytes could allow higher biological protection through additional cell divisions before cells with critically short telomeres undergo apoptosis or replicative senescence compared to males [27]. Interestingly, given that FA supplementation plays a critical role in foetal development for DNA synthesis and cell proliferation, this study raises new questions about the potentially harmful effect of high FA supplementation dosages on embryonic telomerase levels that deserve further investigation.

FAs have a high bioavailability and are quickly absorbed across the intestine [28]. Since they are usually recognised as not harmful to human bodies, a medical prescription is not required for their purchase. Unfortunately, different studies have described that there is unmetabolised FA in the blood of many individuals and that high dosages of FA not metabolised may change some gene expression during cell division over time [3,6,29]. NTD, neoplastic disease, carcinogenic, rheumatoid arthritis, psoriasis, cardiovascular disease, stroke, psychiatric pathologies, and, according to our findings, short TL have been described as the possible consequences of dysregulations of folic acid metabolism [6,29]. In this regard, even if the upper limit dose is unknown, 1 mg/day is considered safe [30]. For its part, the association between excess FA supplementation and TL shortening is a relatively unexplored area in scientific research. However, existing evidence suggests that the mechanism behind the potential deleterious effect of excessive FA intake may be linked to the inhibition of crucial enzymes within the folate pathway [31]. Various folate derivatives have been shown to inhibit these enzymes, including thymidylate synthase, methylenetetrahydrofolate dehydrogenase (MTHFD), 5,10-methenyl tetrahydrofolate cyclohydrolase (which is also performed by MTHFD), and dihydrofolate reductase [3,6,31,32,33]. When folic acid intake exceeds the metabolic capacity of the cell, it can lead to the accumulation of folate derivatives [3,6,32,33]. Inhibition of enzymes, such as MTHFD, dihydrofolate reductase, and thymidylate, synthase results in reduced availability of nucleotide precursors, particularly thymidine, essential for DNA synthesis [3,6,32,33]. This shortage in nucleotide precursors might lead to DNA damage, which, in turn, may contribute to the shortening of telomeres [31]. While this hypothesis warrants further investigation, it might shed light on a potential mechanism underlying the association between excess FAs and TL shortening.

Contemplating the consequences mentioned above, our findings are highly relevant considering the high consumption of this micronutrient during pregnancy, and in many cases, without the correct supervision. Therefore, future studies must investigate the over-consumption of this micronutrient through supplementation, considering that numerous countries additionally supplement their foods with FA. Understanding the potential implications of excess FA intake is crucial, not only for maternal and child health but also for informing public health policies and clinical guidelines. Given the widespread availability and use of FAs, further research can help refine recommendations, ensuring that they align with the latest scientific evidence.

### Strengths and Limitations

This study leveraged data from the INMA birth cohort study, a pioneer project in Spain investigating the role of environmental factors during pregnancy and the beginning of life on growth and development. TL was objectively measured following standard methods by trained professionals. In addition, we adjusted our analyses for an extensive range of confounding factors, including data from the mothers and children at age four. However, this study is not exempt from limitations. First, although children from the INMA project were from different Spain areas, they may not represent the Spanish child population; therefore, estimates should not be fully generalised. Second, FAs were self-reported, which may cause some inaccuracies. We used a validated FFQ, which showed a satisfactory correlation of 0.53 when dietary folate and FA use were correlated with folate serum levels in a subsample of the pregnant women in the Valencia cohort. Third, LTL was measured using PCR, which shows a higher technical variability than, e.g., terminal restriction fragment (TRF) analysis. However, in large cross-sectional settings, as assessed by qPCR, LTL may be in line with the TRF-estimated LTL [34]. Furthermore, among the limitations of the PCR method is that it does not provide absolute LTL measures, as well as issues in detecting very short telomeres or telomeric losses. Therefore, even if a large amount of epidemiological research on TL has been conducted using the PCR approach [35], findings should be interpreted cautiously, and telomere dynamics should be confirmed in future longitudinal-based studies. Fourthly, unmeasured or residual confounding is possible even if we included a long list of confounding factors. Finally, because of the observational nature of this study, causality cannot be inferred. Nonetheless, the prospective design of the INMA project allows long-term effects in follow-up assessments to be verified and potential etiological factors of disturbances of normal child development over time to be identified, thereby establishing a temporal sequence of events.

## 5. Conclusions

This study shows that children whose mothers were supplemented with high dosages of FAs during the first period, as well as during the whole period of pregnancy, may have shorter TL at age four, independent of a wide range of confounding factors. When the analyses were stratified by sex, boys—but not girls—were those who presented the strongest associations and shorter telomeres at the same age. In this context, and considering the high intake of this nutrient during this period, future studies still need to elucidate what may be the correct dosages pregnant women should take, or if the supplementation is necessary in all cases. Moreover, we still need to understand further the biological pathways that might explain the association between TL and folic acid supplements.

## Figures and Tables

**Table 1:** Sociodemographic and lifestyle characteristics of the population included by area of origin.

Children’s Characteristics	All Areas	Asturias	Gipuzkoa	Sabadell	*p*
*n* (%)	666 (100)	264 (39.6)	132 (19.8)	270 (40.6)	-
Sex, *n* (%)					0.523
Girls	317 (47.6)	120 (45.5)	68 (51.5)	129 (47.8)	
Boys	349 (52.4)	144 (54.5)	64 (48.5)	141 (52.2)	
Season of blood extraction at age four, *n* (%)					<0.001
Winter	179 (26.9)	97 (36.8)	8 (6.1)	74 (27.4)	
Spring	223 (33.5)	71 (26.9)	73 (55.3)	79 (29.3)	
Summer	102 (15.3)	26 (9.8)	25 (18.9)	51 (18.9)	
Autumn	162 (24.3)	70 (26.5)	26 (19.7)	66 (24.4)	
Relative Mediterranean diet score, median (IQR)	9.0 (7.0; 10.0)	9.0 (8.0; 11.0)	8.0 (7.0; 10.0)	8.0 (6.0; 10.0)	<0.001
TV time, (h/week), median (IQR)	9.0 (5.6; 13.5)	9.0 (5.8; 12.0)	7.0 (4.5; 11.5)	10 (6.5; 14.0)	<0.001
Sleep (h/day), median (IQR)	10.5 (10.0; 11.0)	10.5 (10.0; 11.0)	11.0 (10.0; 11.0)	10.4 (10.0; 11.0)	0.104
UPF intake (g/day), median (IQR)	390.6 (280.5; 553.5)	314.4 (228.4; 433.7)	382.6 (277.1; 562.8)	481.5 (352.2; 633.3)	<0.001
**Mother’s characteristics**					
Age (years), median (IQR)	31 (29; 34)	32 (29; 35)	32 (29; 34)	30 (28; 33)	<0.001
Parity, *n* (%)					0.110
0	380 (57.1)	163 (61.7)	68 (51.5)	149 (55.2)	
≥1	286 (42.9)	101 (38.3)	64 (48.5)	121 (44.8)	
Smoking status (yes), *n* (%)	159 (23.9)	65 (24.6)	21 (15.9)	73 (27.0)	<0.001
Educational level, *n* (%)					<0.001
Primary	131 (19.7)	41 (15.5)	20 (15.1)	70 (25.9)	
Secondary	274 (41.1)	120 (45.5)	41 (31.1)	113 (41.9)	
University	261 (39.2)	103 (39.0)	71 (53.8)	87 (32.2)	
Folate intake (1st period)	308.4 (262.5; 353.0)	313.3 (264.4; 366.8)	336.8 (296.0; 376.0)	285.0 (238.8; 329.6)	<0.001
Folate intake (2nd period)	299.2 (255.2; 352.3)	304.4 (258.4; 358.7)	341.7 (287.9; 377.8)	281.2 (242.2; 318.3)	<0.001
Folate intake (whole period)	302.1 (266.4; 346.0)	311.5 (267.4; 354.4)	228.0 (294.7; 375.3)	282.6 (252.6; 322.6)	<0.001
Energy intake (1st period)	1986 (1718; 2306)	2019 (1748; 2310)	2870 (1608; 2134)	2028 (1775; 2331)	<0.001
Energy intake (2nd period)	1984 (1687; 2315)	1947 (1644; 2330)	1924 (1603; 2152)	2067 (1801; 2393)	<0.001
Energy intake (whole period)	2000 (1772; 2269)	1990 (1782; 2280)	1903 (1625; 2124)	2062 (1798; 2311)	<0.001
Alcohol intake (1st period)	0 (0; 0.04)	0 (0; 0)	0 (0; 0.05)	0 (0; 0.09)	<0.001
Alcohol intake (2nd period)	0 (0; 0.09)	0 (0; 0.04)	0.04 (0; 0.3)	0 (0; 0.3)	<0.001
Alcohol intake (whole period)	0 (0; 0.13)	0 (0; 0.04)	0.04 (0; 0.18)	0.2 (0; 0.3)	<0.001

IQR, interquartile range; *n*, number; h, hour; UPF, ultra-processed food.

**Table 2:** Association between folic acid supplementation categories during three pregnancy periods and telomere length at age four.

Folic Acid Supplementation	First Period ^a^				Second Period ^b^				Whole Period ^c^			
	*n*	% Difference (95% CI)	*p*	*I* ^2^	*n*	% Difference (95% CI)	*p*	*I* ^2^	*n*	% Difference (95% CI)	*p*	*I* ^2^
<400 μg/d	356	Ref.	-	-	383	Ref.	-	-	371	Ref.	-	-
≥400 to 999 μg/d	97	2.70 (−5.99; 11.4)	0.543	69.9	149	−2.55 (−6.72; 1.62)	0.231	0.0	113	1.11 (−5.13; 2.90)	0.586	0.0
≥1000 to 4999 μg/d	157	0.23 (−3.19; 3.65)	0.896	16.3	44	−3.16 (−9.00; 2.69)	0.290	0.0	161	1.40 (−2.35; 5.16)	0.464	0.0
≥5000 μg/d	56	−6.07 (−12.60; 0.46)	0.068	0.0	90	−2.10 (−8.15; 3.96)	0.497	0.0	21	−13.5 (−27.4; 0.35)	0.055	69.4
	***n***	**% difference ^d^ (95% CI)**	***p***	***I* ^2^**	***n***	**% difference ^d^ (95% CI)**	***p***	***I* ^2^**				
<400 μg/d	356	Ref.	-	-	383	Ref.	-	-				
≥400 to 999 μg/d	97	2.73 (−5.83; 11.3)	0.532	69.2	149	−2.72 (−6.86; 1.42)	0.198	0.0				
≥1000 to 4999 μg/d	157	0.02 (−4.23; 4.27)	0.993	37.6	44	−1.71 (−7.92; 4.50)	0.590	0.0				
≥5000 μg/d	56	−7.28 (−14.42; −0.13)	0.046	0.0	90	0.93 (−5.68; 7.54)	0.784	0.0				

Data are presented as % difference and their respective 95% CI by gestational period and folic acid supplementation categories using multiple regression analyses. All analyses were adjusted for mother’s age, parity, smoking status, education level, intake of alcohol, folate, and total energy intake (according to each of the periods included: first, second or all); sex of the child, blood extraction date; and sleep, ultra-processed food intake, rMED, and TV time of the child at age four. ^a^ The first period (periconceptional period) of pregnancy covers the months from preconception to the first trimester of pregnancy; ^b^ the second period (third trimester) of pregnancy covers the months from the first to the third trimester of pregnancy; ^c^ the whole period of pregnancy covers the months from preconception to the third trimester of pregnancy; ^d^ the folic acid supplementation in the first and the second period were mutually adjusted.

**Table 3:** Association between folic acid supplementation categories during three pregnancy periods and telomere length at age four in girls.

Folic Acid Supplementation	First Period ^a^				Second Period ^b^				Whole Period ^c^			
	*n*	% Difference (95% CI)	*p*	*I* ^2^	*n*	% Difference (95% CI)	*p*	*I* ^2^	*n*	% Difference (95% CI)	*p*	*I* ^2^
<400 μg/d	172	Ref.	-	-	186	Ref.	-	-	184	Ref.	-	-
≥400 to 999 μg/d	48	−1.30 (−9.23; 6.63)	0.748	0.0	67	−4.10 (−10.1; 1.94)	0.183	0.0	45	−5.44 (−12.1; 1.27)	0.112	0.0
≥1000 to 4999 μg/d	68	0.86 (−5.77; 7.48)	0.800	0.0	23	1.81 (−7.86; 11.5)	0.713	0.0	77	2.10 (−3.66; 7.87)	0.474	0.0
≥5000 μg/d	29	−2.32 (−15.3; 10.7)	0.726	0.0	41	1.55 (−6.77; 9.86)	0.715	16.2	11	0.01 (−0.06; 0.07)	0.799	0.0
	***n***	**% difference ^d^ (95% CI)**	***p***	***I*** **^2^**	***n***	**% difference ^d^ (95% CI)**	***p***	***I*** **^2^**				
<400 μg/d	172	Ref.	-	-	186	Ref.	-	-				
≥400 to 999 μg/d	48	2.46 (−4.50; 9.41)	0.489	46.6	67	−4.11 (−10.3; 2.11)	0.196	0.0				
≥1000 to 4999 μg/d	68	−1.71 (−10.2; 6.84)	0.700	0.0	23	3.54 (−6.33; 13.4)	0.483	0.0				
≥5000 μg/d	29	−5.42 (−17.2; 6.39)	0.368	0.0	41	2.82 (−5.70; 11.3)	0.516	0.0				

Data are presented as % difference and their respective 95% CI by gestational period and folic acid supplementation categories using multiple regression analyses. All analyses were adjusted for mother’s age, parity, smoking status, education level, intake of alcohol, folate, and total energy intake (according to each of the periods included: first, second or all); blood extraction date; and sleep, ultra-processed food intake, rMED, and TV time of the child at age four. ^a^ The first period (periconceptional period) of pregnancy covers the months from preconception to the first trimester of pregnancy; ^b^ the second period (third trimester) of pregnancy covers the months from the first to the third trimester of pregnancy; ^c^ the whole period of pregnancy covers the months from preconception to the third trimester of pregnancy; ^d^ The folic acid supplementation in the first and the second period was mutually adjusted.

**Table 4:** Association between folic acid supplementation categories during three pregnancy periods and telomere length at age four in boys.

Folic Acid Supplementation	First Period ^a^				Second Period ^b^				Whole Period ^c^			
	*n*	% Difference (95% CI)	*p*	*I* ^2^	*n*	% Difference (95% CI)	*p*	*I* ^2^	*n*	% Difference (95% CI)	*p*	*I* ^2^
<400 μg/d	184	Ref.	-	-	197	Ref.			187	Ref.	-	-
≥400 to 999 μg/d	49	0.30 (−7.35; 7.95)	0.939	0.0	82	0.01 (−6.50; 6.52)	0.998	0.0	68	3.23 (−2.38; 8.85)	0.259	0.0
≥1000 to 4999 μg/d	89	−0.20 (−4.47; 4.07)	0.928	12.0	21	−7.98 (−15.4; −0.53)	0.036	0.0	84	0.36 (−4.55; 5.27)	0.886	0.0
≥5000 μg/d	27	−11.3 (−19.4; −3.22)	0.006	22.2	49	8.28 (−8.84; 25.4)	0.343	54.3	10	−7.84 (−22.5; 6.85)	0.295	56.1
	***n***	**% difference ^d^ (95% CI)**	***p***	***I*** **^2^**	***n***	**% difference ^d^ (95% CI)**	***p***	***I*** **^2^**				
<400 μg/d	184	Ref.	**-**	**-**	197	Ref.	**-**	**-**				
≥400 to 999 μg/d	49	0.45 (−7.27; 8.17)	0.909	0.0	82	0.18 (−6.18; 6.55)	0.955	0.0				
≥1000 to 4999 μg/d	89	0.36 (−5.11; 5.83)	0.898	10.5	21	−7.97 (−15.9; −0.04)	0.049	0.0				
≥5000 μg/d	27	−13.5 (−23.0; −4.04)	0.005	0.0	49	4.55 (−5.46; 14.6)	0.373	21.4				

Data are presented as % difference and their respective 95% CI by gestational period and folic acid supplementation categories using multiple regression analyses. All analyses were adjusted for mother’s age, parity, smoking status, education level, intake of alcohol, folate, and total energy intake (according to each of the periods included: first, second or all); blood extraction date; and sleep, ultra-processed food intake, rMED, and TV time of the child at age four. ^a^ The first period (periconceptional period) of pregnancy covers the months from preconception to the first trimester of pregnancy; ^b^ the second period (third trimester) of pregnancy covers the months from the first to the third trimester of pregnancy; ^c^ the whole period of pregnancy covers the months from preconception to the third trimester of pregnancy; ^d^ the folic acid supplementation in the first and the second period were mutually adjusted.

## Data Availability

Not applicable.
